# Supplementary material for: Impact of Prior Antibiotic Use in Primary Care on Escherichia coli Resistance to Third Generation Cephalosporins: A Case-Control Study
Source: Antibiotics (Basel). 2021 Apr 16;10(4):451. doi: 10.3390/antibiotics10040451 (PMC8073604; doi:10.3390/antibiotics10040451)
Supplement: Supplementary file 1 [file antibiotics-10-00451-s001.zip › antibiotics-1165243-supplementary.pdf]

# supplementary file

**Table S1.** Codes for exposure

| ATC | 3GC |
|---|---|
| J01DD01 | Cefotaxime |
| J01DD02 | Ceftazidime |
| J01DD04 | Ceftriaxone |
| J01DD08 | Cefixime |
| J01DD13 | Cefpodoxime |

**Abbreviations**. ATC = Anatomical therapeutic class; 3GC = third generation cephalosporin

**Table S2.** Codes for considered comorbidities

| Comorbidity | ATC | ICD-9-CM |
|---|---|---|
| Cancer | L01, L02A, L02B | 140, 208 |
| Diabetes | A10 | 250 |
| COPD | R03BB | 490, 491.1, 491.20, 491.21, 491.22, 491.8, 491.9, 492.0, 492.8, 493.22, 496 |
| AIDS | J05A | 42 |
| Immunosuppression | | 279 (except 279.4) |
| Haemodialysis | | 399.5, 549.8, 392.7 |

**Abbreviations**. ATC = Anatomical therapeutic class; ICD-9-CM = International Classification of Diseases, Ninth Revision, Clinical Modification, AIDS = Acute immune deficiency syndrome; COPD = Chronic obstructive pulmonary disorder.

**Table S3**. Sensitivity analysis 1 (excluded patients tested 48 hours after hospital admission)

| Variables | Cases[a] (N=74) | Controls[a] (N=477) | Adj OR (95% CI) | p |
|---|---|---|---|---|
| Age, Median (IQ) | 81.5 (74 – 87) | 78 (63 – 86) | 1.27[b](1.01 – 1.60) | 0.038 |
| Gender, Male (%) | 30 (4.54) | 147 (30.82) | 2.78 (1.25 – 6.17) | 0.012 |
| Drug's DDD taken in previous 5 years, Median (IQ) | 30 (4.54) | 147 (30.82) | 2.78[c](1.25 – 6.17) | 0.012 |
| Number of antibiotics taken in previous 5 years, Median (IQ) | 3 (1 – 4) | 2 (1 – 3) | 1.28 (1.02 – 1.60) | 0.030 |
| At least one other J01 prescription in previous year (%) | 50 (67.57) | 202 (42.35) | 1.33 (0.58 – 3.04) | 0.494 |
| 3GC prescription in previous year (%) | | | | |
| 0 | 64 (86.49) | 437 (91.61) | - | - |
| 1 | 4 (5.41) | 30 (6.29) | 0.47 (0.11 – 2.01) | 0.306 |
| 2+ | 6 (8.11) | 10 (2.10) | 2.79 (0.63 – 12.27) | 0.175 |
| Hospitalization days, Median (IQ) | 56.5 (16 – 118) | 14 (0 – 53) | 1.06[d](0.99 – 1.12) | 0.057 |
| Hospitalization with surgery (%) | 37 (50.00) | 182 (38.16) | 0.92 (0.75 – 1.14) | 0.471 |
| Diabetes (%) | 23 (31.08) | 83 (17.40) | 1.57 (0.66 – 3.72) | 0.307 |

[a]Number (%) of patients or median (IQ); [b]OR calculated for 10-year increments; [c]OR calculated for 1000-DDD increments; [d]OR calculated for 10-day increments

**Abbreviations**. IQ = Interquartile range; DDD = Defined daily dose, 3GC = third-generation cephalosporin

**Table S4**. Sensitivity analysis 2 (excluded patients who received a 3GC-prescription 15 days prior to hospitalization)

| Variables | Cases[a] (N=239) | Controls[a] (N=1546) | Adj OR (95% CI) | p |
|---|---|---|---|---|
| Age, Median (IQ) | 79 (68 – 85) | 76 (61 – 84) | 1.11[b](1.02 – 1.21) | 0.012 |
| Gender, Male (%) | 106 (44.35) | 525 (33.96) | 1.56 (1.15 – 2.11) | 0.004 |
| Drug's DDD taken in previous 5 years, Median (IQ) | 4334.15 (1075.42 – 7914.43) | 3155.58 (414.83 – 6416.38) | 0.95[c](0.91 – 0.99) | 0.010 |
| Number of antibiotics taken in previous 5 years, Median (IQ) | 3 (1 – 4) | 2 (0 – 3) | 1.15 (1.05 – 1.25) | 0.002 |
| At least one other J01 prescription in previous year (%) | 154 (64.44) | 634 (41.01) | 2.02 (1.44 – 2.83) | <0.0001 |
| 3GC prescription in previous year (%) | | | | |
| 0 | 212 (88.70) | 1437 (92.95) | - | - |
| 1 | 11 (4.60) | 77 (4.98) | 0.68 (0.34 – 1.36) | 0.277 |
| 2+ | 16 (6.69) | 32 (2.07) | 2.56 (1.27 – 5.14) | 0.008 |
| Hospitalization days, Median (IQ) | 8 (47 – 100) | 12 (0 – 45) | 1.06[d](1.04 – 1.08) | <0.0001 |
| Hospitalization with surgery (%) | 116 (48.54) | 637 (41.20) | 0.90 (0.83 – 0.98) | 0.014 |
| Diabetes (%) | 61 (25.52) | 250 (16.17) | 1.59 (1.09 – 2.32) | 0.017 |

[a]Number (%) of patients or median (IQ); [b]OR calculated for 10-year increments; [c]OR calculated for 1000-DDD increments; [d]OR calculated for 10-day increments

**Abbreviations**. IQ = Interquartile range; DDD = Defined daily dose, 3GC = third-generation cephalosporin

**Table S5**. Sensitivity analysis 3 (included controls where those with *E.coli* sensitive to any antibiotic)

| Variables | Cases[a] (N=241) | Controls[a] (N=835) | Adj OR (95% CI) | p |
|---|---|---|---|---|
| Age, Median (IQ) | 79 (68 – 85) | 75 (57 – 84) | 1.09[b](0.99 – 1.21) | 0.054 |
| Gender, Male (%) | 106 (43.98) | 264 (31.62) | 1.43 (1.01 – 2.04) | 0.046 |
| Drug's DDD taken in previous 5 years, Median (IQ) | 4298.0 (1079.0 – 7792.1) | 2828.5 (236.8 – 5719.3) | 0.94[c](0.89 – 0.99) | 0.017 |
| Number of antibiotics taken in previous 5 years, Median (IQ) | 3 (1 – 4) | 1 (0 – 3) | 1.31 (1.13 – 1.51) | <0.0001 |
| At least one J01 prescription taken in previous 5,4,3 years (%) | 161 (66.80) | 444 (57.17) | 0.66 (0.42 – 1.05) | 0.080 |
| At least one J01 prescription taken in previous 2 year (%) | 127 (52.70) | 282 (33.77) | 1.14 (0.74 – 1.75) | 0.556 |
| At least one J01 prescription in previous year (%) | 161 (66.80) | 315 (37.72) | 2.08 (1.41 – 3.08) | <0.0001 |
| Hospitalization days, Median (IQ) | 47 (8 – 97) | 8 (0 – 33) | 1.12[d](1.08 – 1.15) | <0.0001 |
| Hospitalizations with surgery (%) | 117 (48.55) | 313 (37.49) | 0.88 (0.79 – 0.98) | 0.021 |
| Diabetes (%) | 61 (25.31) | 115 (13.77) | 1.74 (1.09 – 2.79) | 0.020 |

[a]Number (%) of patients or median (IQ); [b]OR calculated for 10-year increments; [c]OR calculated for 1000-DDD increments; [d]OR calculated for 10-day increments

**Abbreviations**. IQ = Interquartile range; DDD = Defined daily dose

**Table S6**. Sensitivity analysis 4 (included controls where those with *E.coli* sensitive to any antibiotic)

| Variables | Cases[a] (N=241) | Controls[a] (N=835) | Adj OR (95% CI) | p |
|---|---|---|---|---|
| Age, Median (IQ) | 79 (68 – 85) | 75 (57 – 84) | 1.10[b] (1.00 – 1.21) | 0.046 |
| Gender, Male (%) | 106 (43.98) | 264 (31.62) | 1.45 (1.02 – 2.06) | 0.040 |
| Drug's DDD taken in previous 5 years, Median (IQ) | 4298.0 (1079.0 – 7792.1) | 2828.5 (236.8 – 5719.3) | 0.93[c](0.89 – 0.98) | 0.008 |
| Number of antibiotics taken in previous 5 years, Median (IQ) | 3 (1 – 4) | 1 (0 – 3) | 1.23 (1.10 – 1.37) | <0.0001 |
| At least one other J01 prescription in previous year (%) | 3 (1 – 4) | 1 (0 – 3) | 1.23 (1.10 – 1.37) | <0.0001 |
| 3GC prescription in previous year (%) | | | | |
| 0 | 3 (1 – 4) | 1 (0 – 3) | 1.23 (1.10 – 1.37) | <0.0001 |
| 1 | 12 (4.98) | 36 (4.31) | 0.83 (0.37 – 1.88) | 0.661 |
| 2+ | 17 (7.05) | 14 (1.68) | 2.27 (0.98 – 5.27) | 0.056 |
| Hospitalization days, Median (IQ) | 47 (8 – 97) | 8 (0 – 33) | 1.11[d](1.08 – 1.15) | <0.0001 |
| Hospitalization with surgery (%) | 117 (48.55) | 313 (37.49) | 0.88 (0.79 – 0.99) | 0.031 |
| Diabetes (%) | 61 (25.31) | 115 (13.77) | 1.82 (1.14 – 2.90) | 0.012 |

[a]Number (%) of patients or median (IQ); [b]OR calculated for 10-year increments; [c]OR calculated for 1000-DDD increments; [d]OR calculated for 10-day increments

**Abbreviations**. IQ = Interquartile range; DDD = Defined daily dose, 3GC = third-generation cephalosporin
